# GIS-based evaluation of groundwater geochemistry and statistical determination of the fate of contaminants in shallow aquifers from different functional areas of Agra city, India: levels and spatial distributions

**DOI:** 10.1039/c8ra00577j

**Published:** 2018-04-27

**Authors:** Krishna Kumar Yadav, Neha Gupta, Vinit Kumar, Priya Choudhary, Shakeel Ahmad Khan

**Affiliations:** Institute of Environment and Development Studies, Bundelkhand University Jhansi, 284128 India envirokrishna@gmail.com +91-9473949343; Centre for Environment Science and Climate Resilient Agriculture, Indian Agricultural Research Institute New Delhi, 110012 India

## Abstract

The quality of groundwater is very important in Agra because groundwater is the main source of water for drinking, domestic, agricultural and industrial uses. A groundwater geochemistry study was conducted in Agra where 28 samples were collected from shallow aquifers in May 2016 from different sites. The aim of this research was to assess the quality of groundwater for drinking purposes in the study area. Arc-GIS has been used to prepare geographic information system-based spatial distribution maps of different major elements. The groundwater quality was analyzed for various physico-chemical parameters, major cations and anions and some trace metals. The observed values were compared with BIS and WHO standards. Statistical parameters such as the mean, median, standard deviation, skewness and kurtosis were used to analyze the hydrogeochemical characteristics of the groundwater. Correlation coefficient analysis and principal component analysis (PCA) were performed to identify the sources of the water constituents. Our results showed that most of the samples exceeded the acceptable limit for drinking water standards. The sequence of abundance of the main cations was generally Na^+^ > Ca^2+^ > Mg^2+^ > K^+^, while the anions in order of abundance were HCO_3_^−^ > Cl^−^ > SO_4_^2−^ and NO_3_^−^ > F^−^. All of the trace metals were within the permissible limit except for iron and manganese. The hazard index value of 5.7 × 10^−2^ indicated that there was no potential health risk in the study area. Ca^2+^, Mg^2+^, Cl^−^ and SO_4_^2−^ were the dominant hydrogeochemical facies in the majority of the groundwater samples. Most of the parameters such as TDS, Cl^−^, HCO_3_^−^, SO_4_^2−^, NO_3_^−^, Ca^2+^, Mg^2+^, Na^+^, K^+^ and TH showed strong correlations with each other, which were due to natural processes such as weathering, exchangeable ions and reduction/oxidation, as well as anthropogenic activity around the study area. The water quality index indicated that the water quality was poor at 46.43% of the sampling sites, very poor at 28.57% of the sites and unsuitable for drinking purposes at 25% of the sampling sites. Gibbs diagrams suggested rock weathering as a major driving force for controlling the groundwater chemistry in the study area, along with evaporation as a minor influence.

## Abstract 2

A groundwater geochemistry study was conducted in Agra where 28 samples were collected from shallow aquifers in May 2016 from different sites. The aim of this research was to assess the quality of groundwater for drinking purposes in the study area.

## Graphical abstract

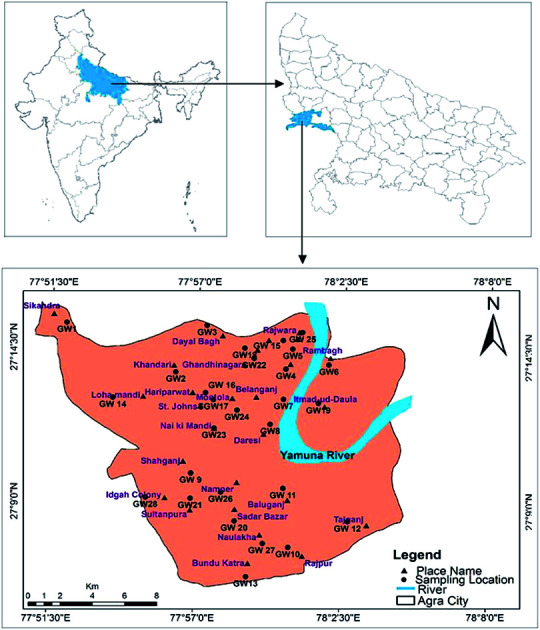

## 1. Introduction

The quality of water is a vital concern for mankind as it is directly linked to human welfare. Groundwater, rivers, streams and wells are usual sources of drinking water which is usually untreated.^1,2^ More than 90% of the Indian population from several states rely on groundwater for drinking and other purposes.^3,4^ However, the indiscriminate use of chemical fertilizers, insecticides and pesticides, the improper disposal of waste, and chemical spills from industry have caused a deterioration in groundwater quality.^5^ Landfill leachate is also a significant source of groundwater pollution.^6^ Water quality is an important worldwide environmental issue and it involves a large number of physicochemical parameters, including heavy metals, anions and cations present in the groundwater.^7^ Heavy metal contamination is of great concern due to the toxicity, persistence and bioaccumulation of heavy metals. The accumulation of heavy metals above the threshold level is mainly due to anthropogenic activities including mining, chemical manufacturing and agriculture, and from hospital wastewater and electronic waste.^8^ Metals like copper, iron, manganese and zinc are essential for life processes, whereas others such as cadmium, nickel and mercury have no physiological functions but often result in harmful disorders at higher concentrations.^9–11^ Mercury toxicity in humans can cause nervous, respiratory and renal damage. It is more toxic in its organic form, *i.e.* methyl mercury, when consumed or inhaled, while cadmium is highly toxic to the kidneys. Chronic exposure to arsenic may adversely affect the cardiovascular, renal, pulmonary, gastrointestinal, hepatic, neurological, reproductive and respiratory systems. It may also cause cancer in humans.^12^ Lead is one of the most toxic heavy metals that disturbs physiological processes in living beings.^13^ Cr(vi) is also toxic to humans, while its reduced form, Cr(iii), does not act as an essential contaminant in groundwater.^14^ Groundwater chemistry provides a better understanding of possible alterations in its quality. It also determines its suitability for domestic and irrigation purposes.^15^ A number of studies on groundwater and surface water quality have been carried out in different parts of India and around the world through in terms of major ion chemistry, trace element chemistry and through multivariate statistical techniques. However, the characteristics of groundwater quality in Agra have not been investigated so far using multivariate statistical methodology. Prerna *et al.*^16^ found that the concentration of Fe and Mn was higher than the permissible limit designated by the WHO and BIS in the Agra region. Kumar *et al.*^17^ evaluated the groundwater quality in the Agra district for irrigation purposes using Wilcox and Piper diagrams.

The present study uses statistical tools, including principal component analysis (PCA) and Pearson correlation matrices, to resolve and interpret the complex dataset. On the other hand, the water quality index has been evaluated to assess the drinking water quality and suitability in the area. The hydrochemical facies have been classified with the support of Piper trilinear diagrams to determine the chemical characteristics of groundwater in Agra. The average daily dose and hazard quotient were calculated to assess the health risk associated with the ingestion of trace metals present in groundwater in the study area. However, the objective of this paper is to develop a reliable multi-statistical method to characterize the water quality of groundwater samples in Agra, which will be useful for decision makers to take the proper initiative for groundwater quality management.

## 2. Materials and methods

### 2.1. Study area

**Fig. 1:**
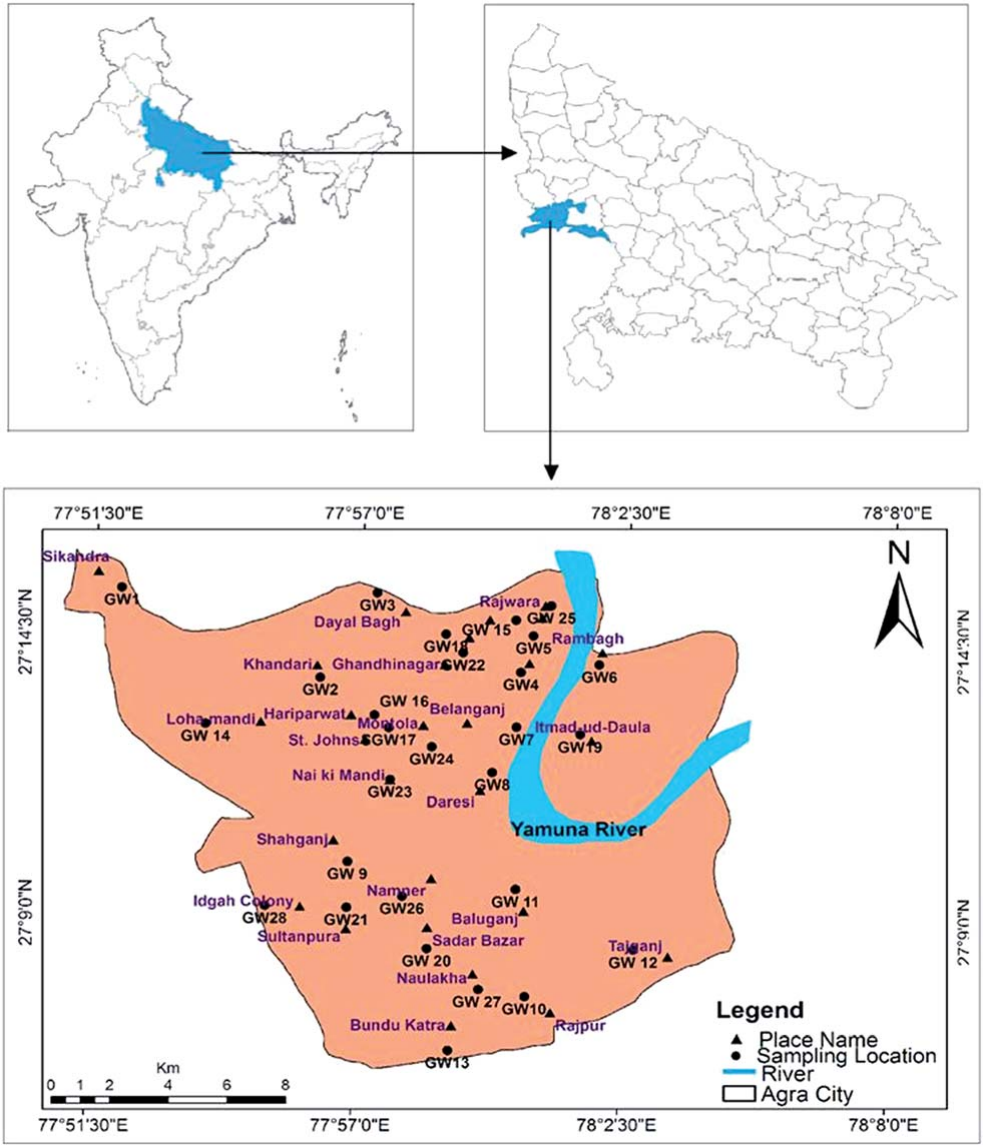
Agra city map showing groundwater sampling locations.

Agra is a city where one of the seven wonders is located, known as the Taj Mahal. The city lies in Western Uttar Pradesh, situated on the banks of the Yamuna river, 185 km southeast of New Delhi. The average elevation of the study area was around 169 m above sea level, and the city lies at 27°10′ N and 78°02′ E, as shown in Fig. 1. The total area of Agra district is 4041 km^2^, of which 279.998 km^2^ of urban area was sampled in this study. According to the national census of 2011, the total population of the city is 4 418 797 (http://upenvis.nic.in/Database/Agra_930.aspx). The city experiences various seasons such as mild winters, dry and hot summers and monsoon seasons. The climate of the city is a semi-arid to subtropical climate. The temperature rises from 21.9 °C to 45 °C in the summer and drops to 4.2 °C in the winter. The mean annual rainfall is 687.2 mm,^18^ 95% of which is expected to come from a southwest monsoon in July to September, with an average evapotranspiration rate of 1466 mm per year. The daily relative humidity varies from 30 to 100%. Agra is a major tourist destination and approximately 7200 small-scale industrial units are also established. The economy is dependent on the industrial sector, which includes automobiles, leather goods, handicrafts and stone carving. There has been rapid exploitation of the groundwater resources during the last decade. Additionally, large scale pollution has occurred due to pressure from the increased industrialization and urbanization and the increase in population.

### 2.2. Hydrogeology of the area

The Agra region occupies a part of the Indo-Gangetic plains with quaternary sediments, which mainly comprise a sequence of clay, silt, different grades of sand, gravel and kankar (CaCO_3_ concretions) in varying proportions.^19,20^ Sedimentary formations were deposited when the valley filled unconformably on the Vindhyan sandstones during the middle to late Pleistocene and Holocene times. These comprise different grades of sand, silt, clay, gravel and secondarily developed calcareous nodules known as kankar. The majority of the region is comprised of quaternary age alluvium. The alluvium was deposited over a base of Vindhyan rocks, *e.g.* sandstone, shale, silt stone, *etc.* Broad horizons of arkosic gravel/coarse sand are present just above the basal formations in the lower part.^20^ Vindhyan rock formations consist of rocks of the Bhander group, which include white to purple quartz arenite, medium to fine-grained purplish to reddish spotted and laminated sandstone with intermittent deposits of shales, shale pebble conglomerate, siltstone and greenish sandstone.^18^ Due to the varied hydrogeochemical conditions and significant dissimilarities in lithologies and climatic conditions, the geological formation is highly diversified, which further complicates the study of groundwater behavior.^21^ Groundwater occurs mostly in the study area in weathered and fractured zones of unconsolidated sediments. The weathered zones are confined, whereas the fractured zones are semi-confined aquifers.^22^ Semi-confined aquifers are the active recharge zones and contain replenishable groundwater resources. The entire area may broadly be classified into two zones: the western part of the area, with a comparatively shallow depth of the water table, and the eastern part of the area along the Yamuna river, with a deeper water table. The depth of groundwater in Agra differs from 17 to 23 m below ground level (bgl), but it may vary nearby the Agra canal and Yamuna river, and in topographic lows.

### 2.3. Collection of water samples

The systematic random method was adopted for the collection of 28 groundwater samples from shallow aquifers *via* existing tube wells or hand pumps based on their availability in the sampling locations cited in the urban area of Agra city. The samples were collected in May 2016. The water from tube wells is used as drinking water without any prior treatment. Hand pumps of 50 m depth were used for the collection of water samples. Depths were determined through interviews with private well owners. The average groundwater table depth in the study area was 20 mbgl according to the Ground Water Department, Uttar Pradesh.^23^ The water samples were collected only after pumping water for at least 30 min from the tube wells, while the hand pumps were operated for 10–20 min prior to the collection of samples. The water was allowed to flow out in order to obtain stabilized values for temperature, pH and DO. Samples with a total volume of 1 L were collected in polypropylene bottles which were previously rinsed twice with deionized water. Separate samples were collected in 25 ml small bottles for the estimation of trace metal content, and they were preserved at pH 2 with 1% HNO_3_. After the collection, the sample bottles were stored in an ice box in the field and taken to the laboratory, where they were kept in a refrigerator at a temperature of 4 °C.

### 2.4. Experimental analysis

The pH, EC and TDS values were measured on-site immediately after the collection of the samples using a portable meter. The remaining parameters were determined within 2 weeks in the laboratory. Turbidity was measured using a multi-meter water checker (Horiba U-10) in Nephelometric units (NTUs). Total hardness (TH) in terms of CaCO_3_, HCO_3_^−^ and Cl^−^ content was analyzed by the volumetric titration method described by the American Public Health Association (APHA).^24^ The average values of three measurements were calculated for each sample. Dissolved oxygen (DO) was determined using a DO data meter (Eutech CyberScan DO 3000). Concentrations of the major cations (including Ca^2+^, Mg^2+^, Na^+^, K^+^) were measured using a flame photometer (JAISBO Microprocessor). Fluoride anion content was determined by the SPADNS method using a UV-vis spectrophotometer (UV-2450, Shimadzu) at 570 nm. Nitrate and sulphate content were also analyzed using a spectrophotometer at 220 nm and 420 nm, respectively. Major trace metal (Zn, Cu, Fe and Mn) content was measured in mg L^−1^ with the use of an atomic absorption spectrophotometer (AA-7000, Shimadzu) in flame mode after calibration of the respective elements with the specific known standards. Statistical analysis was used to apportion the sources of the contaminants in the water, while a geographical information system (GIS) was used to prepare the geochemical distribution maps.

### 2.5. Quality assurance and quality control

Appropriate protocols for well-purging were used and the accuracy of all analyses was measured using externally supplied standards and calibration check standards, with known additions of the standard to samples and reagent blanks. To ensure the precision of the results, three replicas of the samples were analyzed. All reagents were purchased from Merck. The percent relative standard deviation (RSD) was found to be below 10%, which represents the overall precision for all of the assessed samples examined at the Centre for Environment Science and Climate Resilient Agriculture, Indian Agricultural Research Institute (IARI), New Delhi.

### 2.6. Quantitative health risk assessment

Human exposure to trace metals could occur through three pathways, including oral ingestion, inhalation through the nose and dermal absorption through the skin. The health risks associated with the ingestion of trace metals present in groundwater were assessed using the average daily dose and hazard quotient parameters. The ADD for each trace metal was calculated using eqn (1) adapted from USEPA:^25^1
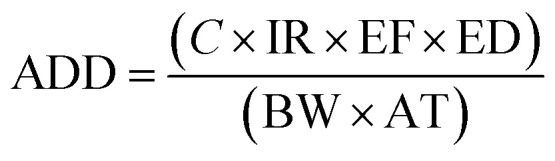
where ADD is the average daily dose (mg per kg per day), *C* is the average concentration of the trace metal in groundwater (mg L^−1^), IR is the ingestion rate (2 L per day), EF is the exposure frequency (365 days per year), ED is the exposure duration (70 years), BW is the body weight (70 kg) and AT is the average time (EF × ED).

The hazard quotient (HQ) for the potential non-carcinogenic risk from each trace metal was determined by dividing the calculated ADD by the reference dose (RfD) using eqn (2):2
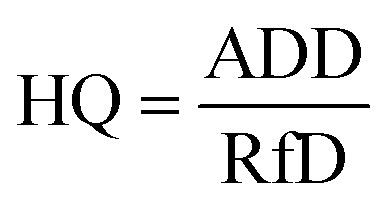
where RfD is the oral toxicity reference dose (mg per kg per day). The value of the RfD for each trace metal was obtained from USEPA.^26^ HQ < 1 is considered to be safe and non-carcinogenic for human health, but HQ > 1 may be a major potential health concern.

The overall potential non-carcinogenic risk posed by all metals was assessed by adding their respective HQ values using eqn (3). The sum of the HQ values of all metals was termed the hazard index (HI). A value of HI > 1 is assumed to have a potential adverse effect on human health.^27^

3 HI = HQ_Zn_ + HQ_Cu_ + HQ_Fe_ + HQ_Mn_

### 2.7. Water quality index (WQI) for groundwater quality

Water quality index is a very useful, effective and efficient tool to communicate information on the overall quality of water.^28^ The estimation of the WQI helps in determining the suitability of groundwater for drinking purposes. Many authors and organizations employ the WQI to meet specific requirements and to express the condition of water.^29–32^ The index reduces large datasets to a single value, facilitating the understanding of the information. The method used for the calculation of the WQI was adapted from Sharma *et al.*^33^ A total of 15 parameters (pH, turbidity, TDS, F^−^, Cl^−^, NO_3_^−^, SO_4_^2−^, HCO_3_^−^, Ca^2+^, Mg^2+^, total hardness, Zn, Cu, Fe and Mn) were considered to calculate the WQI. Each parameter was assigned a definite weight (w_*i*_) according to its relative importance on the overall quality of water, ranging from 1 to 5 (Table 6), where 5 was considered most significant while 1 was least significant. In the second step, the relative weight (*W*_*i*_) was computed using eqn (4):4
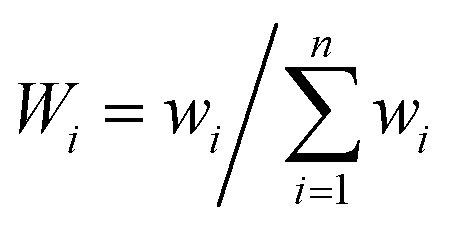
where *W*_*i*_ is the relative weight, *w*_*i*_ is the weight of each parameter and *n* is the number of parameters.

In the next step, the quality rating scale (*q*_*i*_) was measured by comparing the concentration of each parameter in the sample with its respective standard value, as suggested in the BIS guidelines:5
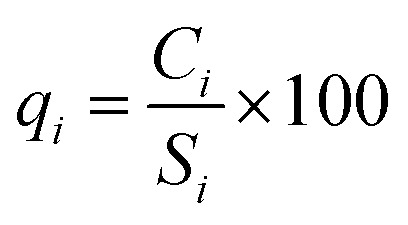
where *q*_*i*_ is the quality rating scale, *C*_*i*_ is the measured concentration of each parameter in mg L^−1^, and *S*_*i*_ is the standard value for each parameter according to BIS^34^ in mg L^−1^.

Sub-indices (SI) were calculated to compute the WQI in the next step using eqn (6).

6 SI_*i*_ = *W*_*i*_ × *q*_*i*_

In final step, the WQI was calculated using eqn (7).

7 WQI = ∑SI_*i*_

### 2.8. Statistical analysis

The mean, range, median, standard deviation, skewness, coefficient of variation, kurtosis and correlation coefficient for different parameters were calculated using Microsoft Excel 2010. The Statistical Package for Social Science (SPSS) software was used for principal component analysis (PCA) and the correlation coefficient was determined in order to identify the sources of different elements in the groundwater sample, as well as inter-element correlation. PCs were extracted by varimax rotation, which selects the variable with the maximum contribution by increasing its participation whilst simultaneously reducing participation of the less contributing variable.

ArcGIS 10.2 software was used to obtain the spatial distribution of the groundwater quality parameters. ArcGIS is a tool which creates layered and spatial maps by analyzing a geographic information database. An inverse distance weighted (IDW) interpolation technique was used for spatial modelling. This technique calculates a value for each grid node by examining the surrounding data points that lie within a user-defined search radius.^35^ All of the data points are used in the interpolation process, and the node value is calculated by averaging the weighted sum of all of the points (Table 1).

**Table 1:** Statistical outline of the measured water parameters with comparison to WHO and Indian standards for drinking water. All parameters are shown in mg L^−1^, except for pH, turbidity and EC, at 25 °C. Turbidity is shown in NTU and EC is shown in μS cm^−1^ a

Parameters	Range	Average	Median	SD	Kurtosis	Skewness	Coefficient of variation	WHO (1997)		BIS (2012)		Percentage of samples exceeding standard limit			
								Minimum desirable	Maximum desirable	Requirement (acceptable limit)	Permissible limit (in the absence of an alternate source)	Minimum desirable (WHO 1997)	Maximum desirable (WHO 1997)	Acceptable limit (BIS 2012)	Permissible limit (BIS 2012)
pH	6.99–7.86	7.42	7.47	0.26	−1.18	−0.04	0.03	7.0–8.5	6.5–9.2	6.5–8.5	No relax.	Nil	Nil	Nil	Nil
Turbidity	2.11–23.43	7.44	5.93	4.86	4.12	1.88	0.67	<5	—	1	5	60.71	—	100	60.71
EC	910–5260	2492.43	2137.5	1215.77	−0.23	0.83	0.48	750	1500	—	—	100	78.57	—	—
TDS	624–3888	1757.43	1531	858.69	0.07	0.89	0.48	500	1500	500	2000	100	50	100	28.57
TH	323.6–1708.63	903.67	851.09	406.26	−0.81	0.49	0.44	100	500	200	600	100	85.71	100	75
DO	1.95–3.94	2.93	2.96	0.55	−0.55	−0.23	0.18	—	—	—	—	—	—	—	—
F^−^	0.9–4.12	1.88	1.85	0.73	2.04	1.32	0.38	0.6–1.5	1.5	1.0	1.5	64.28	64.28	96.42	64.28
Cl^−^	135.95–1155.26	481.75	383.47	278.10	−0.00	0.93	0.57	250	600	250	1000	82.14	28.57	82.14	3.57
HCO_3_^−^	200.5–972.5	497.75	445.37	198.20	0.30	0.92	0.39	200	600	—	—	96.42	21.42	—	—
SO_4_^2−^	48.67–371.5	160.69	121.5	84.03	−0.22	0.73	0.52	200	600	200	400	28.57	Nil	28.57	Nil
NO_3_^−^	9.08–211.83	96.09	86.87	63.16	−1.09	0.43	0.65	—	50	45	No relax.	—	64.28	64.28	—
Ca^2+^	46.5–351.25	164.85	162.87	81.18	0.10	0.65	0.49	75	200	75	200	85.71	32.14	85.71	32.14
Mg^2+^	42.88–363.44	119.59	100.74	61.82	−0.09	0.88	0.51	30	150	30	100	100	21.42	100	50
Na^+^	42.3–598.85	207.41	170.78	130.96	1.61	1.24	0.63	50	200	—	—	96.42	46.42	—	—
K^+^	3.85–68.11	22.45	12.48	19.79	0.13	1.17	0.88	100	200	—	—	Nil	Nil	—	—
Zn	0.016–0.88	0.17	0.082	0.21	3.84	1.93	1.19	3		5	15	Nil		Nil	Nil
Cu	0.00–0.26	0.018	0.00	0.05	20.00	4.30	2.82	2		0.05	1.5	Nil		3.57	Nil
Fe	0.005–1.05	0.32	0.16	0.35	−1.05	0.69	1.08	0.3		0.3	No relax.	39.28		39.28	—
Mn	0.00–0.51	0.08	0.02	0.14	2.21	1.90	1.70	0.1		0.1	0.3	17.85		17.85	14.28

a EC = electrical conductivity, DO = dissolved oxygen, TDS = total dissolved solids, TH = total hardness.

## 3. Results and discussion

### 3.1. Hydrochemistry of the physicochemical parameters

**Fig. 2:**
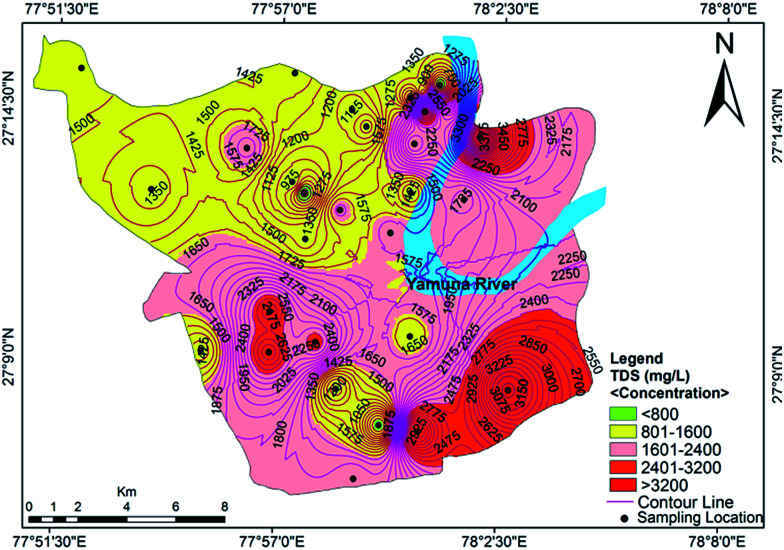
Concentration contour map for TDS showing spatial variation in the groundwater of the study area.

The measured physicochemical parameters are summarized statistically and compared with the WHO and BIS standards in Table 1. The pH values ranging from 6.99 to 7.86, with an average value of 7.42, showed neutral to slightly alkaline dominance in the groundwater of the study area. The turbidity ranged from 2.11 to 23.43 NTU, with an average of 7.44 NTU, where 61% of the water samples exceeded the recommended value of 5 NTU. Drinking water standards do not mandate measurement of dissolved oxygen (DO), but the DO concentration provides meaningful information regarding the stability of many organic and inorganic contaminants in the groundwater.^36^ The mean value of DO concentration was 2.93 mg L^−1^, with minimum and maximum values of 1.95 mg L^−1^ and 3.94 mg L^−1^, respectively. The measured electrical conductivity (EC) ranged from 910 μS cm^−1^ to 5260 μS cm^−1^, where 78% of the samples exceeded the permissible limit designated by the WHO.^37^ High EC values indicate a high ion concentration and/or a high content of dissolved solids in the groundwater. This also signifies multiple a aquifer system and local variation in the soil type.^17^ The value of total dissolved solids (TDS) varied from 624 mg L^−1^ to 3888 mg L^−1^ with an average of 1757 mg L^−1^. The TDS exceeded the desirable limit in 100% of the water samples, but 50% of the samples met the permissible level designated by the WHO^37^ standards for drinking water. The spatial distribution of TDS is shown in Fig. 2. A high spatial variation of EC and TDS is evidence for the heterogeneity of the water chemistry and the involvement of different types of processes. Approximately 75% of the groundwater samples were slightly saline to moderately saline (Table 2) on the basis of groundwater classification and were not suitable for drinking purposes. The high TDS results from the discharge of municipal and industrial effluents, industrial seepage and the percolation of channel water containing solids.

**Table 2:** Groundwater classification on the basis of total dissolved solids (TDS) and total hardness (TH)

S. no.	Class of groundwater	Range of TDS/TH (mg L^−1^)	Samples		Sample number
			No.	%	
**Total dissolved solids (Selvakumar *et al.*** ^**38**^ **)**					
1	Fresh water	<1000	4	14.28	15, 17, 25, 28
2	Slightly saline	1000–3000	21	75	1, 2, 3, 4, 5, 7, 8, 9, 11, 13, 14, 16, 18, 19, 20, 21, 22, 23, 24, 26, 28
3	Moderately saline	3000–10 000	3	10.71	6, 10, 12
4	Very saline	10 000–30 000	Nil	Nil	Nil
5	Brine	>30 000	Nil	Nil	Nil

**Total hardness (Sawyer and McCarty** ^**39**^ **)**					
1	Soft	<75	Nil	Nil	Nil
2	Moderately hard	75–150	Nil	Nil	Nil
3	Hard	150–300	Nil	Nil	Nil
4	Very hard	>300	28	100	All samples

**Fig. 3:**
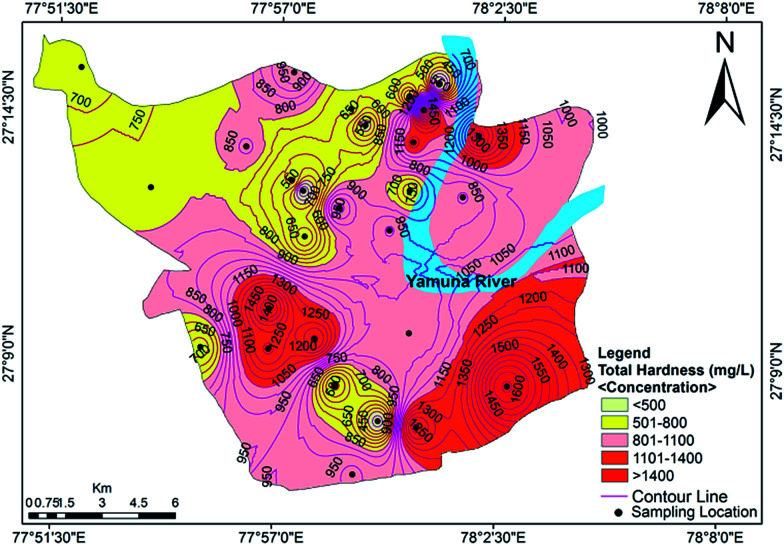
Concentration contour map for the total hardness showing spatial variation in the groundwater of the study area.

Hardness refers to the total concentration of dissolved calcium and magnesium in water. Water is classified as soft, hard, moderately hard and very hard in context of hardness (Sawyer and McCarty^39^). The total hardness (TH) of the analyzed groundwater samples ranged from 323 mg L^−1^ to 1708 mg L^−1^ with a mean value of 903 mg L^−1^. Classification of the groundwater quality in the study area on the basis of hardness content (Table 2) indicated that all of the samples were very hard in nature. The data showed that the hardness of all of the samples exceeded the acceptable limit designated by the BIS and WHO standards, but approximately 25% of samples were under the permissible limit (Fig. 3). Hard water is not desirable for domestic uses because it can cause metal corrosion due to scaly deposition inside pipes, boilers and tanks. It also potentially contributes to a decrease the perceived quality of water, and could pose a danger to human health, causing conditions such as urolithiasis, anencephaly, prenatal mortality, some types of cancer and cardiovascular diseases.^29^

### 3.2. Major anions and cations in groundwater

**Fig. 4:**
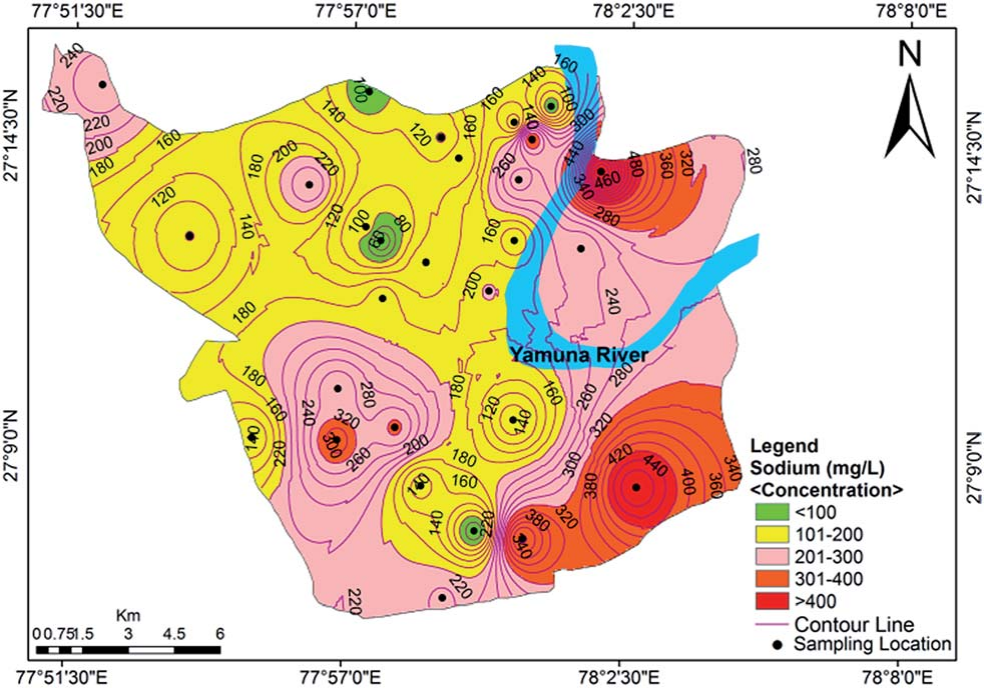
Concentration contour map for sodium showing spatial variation in the groundwater of the study area.

Cation analysis showed that the order of concentration of the cations was Na^+^ > Ca^2+^ > Mg^2+^ > K^+^, with contributions of 40%, 32%, 23% and 5%, respectively. Calcium content varied from a minimum value of 46.50 mg L^−1^ to a maximum value of 351.25 mg L^−1^, with an average of 41.4 mg L^−1^. Approximately 85.71% of the samples exceeded the acceptable limit of 75 mg L^−1^, while 32.14% of the samples exceeded the permissible limit of 200 mg L^−1^. The concentration of Mg^2+^ varied between 42.88 mg L^−1^ and 363.44 mg L^−1^ (avg. 119.59 mg L^−1^). The Ca^2+^ concentration exceeded the Mg^2+^ concentration at many sites, indicating a major supply of limestone, sedimentary rocks and calcium-bearing minerals. A tolerable upper limit is 2500 mg per day for calcium and 350 mg per day for magnesium, above which habitual intake may cause adverse health effects in adults.^40^ The concentrations of Na^+^ and K^+^ ions varied from 42.30 to 598.85 mg L^−1^ (mean value of 207.41 mg L^−1^) and 3.85 to 68.11 mg L^−1^ (mean value of 22.45 mg L^−1^), respectively. Approximately 16% of the samples were observed to have a high concentration of sodium compared to the WHO standards.^37^ A sodium content above the desirable limit can cause hypertension, heart problems, nervous system diseases and kidney diseases.^41^ The spatial distribution map for Na^+^ is shown in Fig. 4. The main sources of potassium in groundwater include rainwater and the weathering of potash and silicate minerals, and there is no recommended standard for the upper level of K^+^ in drinking water.

**Fig. 5:**
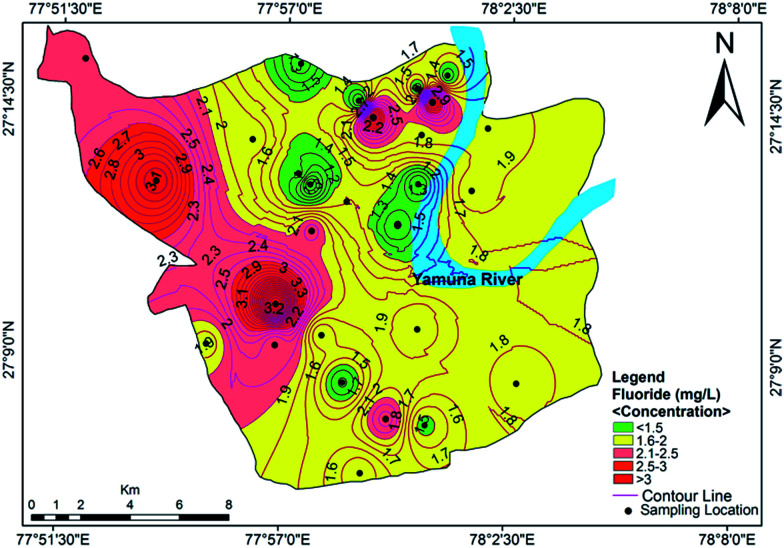
Concentration contour map for fluoride showing spatial variation in the groundwater of the study area.

The anions in order of decreasing concentration were HCO_3_^−^ > Cl^−^ > SO_4_^2−^ > NO_3_^−^ > F^−^, with contributions of 40%, 39%, 13%, 8% and below 1%, respectively. The range of HCO_3_^−^ concentration in the study area was 200.5–972.5 mg L^−1^ with a mean value of 497.75 mg L^−1^. The presence of bicarbonates in soil results from the dissolution of carbonates and silicates by carbonic acid. The chloride concentration was found to be higher than the HCO_3_^−^ concentration, which infers that the dissolution of minerals has taken place in the study area. The chloride content exceeded the desirable limit of 250 mg L^−1^ in 82.14% of the samples, which may impart a noticable salty taste in the groundwater. The higher concentrations of chloride may be due to the weathering of rock, atmospheric deposition, landfill leachates, septic tank effluents, poor sanitary conditions, chemical fertilizers and industrial effluents in sewage.^42^ The concentration of SO_4_^2−^ in the studied samples varied between 48.67–371.5 mg L^−1^, with an average value of 160.69 mg L^−1^. It is ubiquitous in groundwater and does not pose a health risk at the levels normally found in drinking water. However, its higher concentration in drinking water indicates a deteriorating water quality which may cause a health risk. It is commonly derived from the oxidative weathering of sulphide minerals such as pyrite (FeS_2_). However, gypsum and anhydrite are also significant sources of sulphate in water.^43^ The sulphate concentrations were below the permissible limit in all of the investigated samples except for 4, 5, 6, 10, 13 and 21. The nitrate content varied from 9.08 mg L^−1^ to 211.83 mg L^−1^, with a mean value of 96.09 mg L^−1^. About 64.28% of the samples exceeded the WHO guideline level for nitrate in drinking water. Anthropogenic activity, such as septic tanks, seepage beds, municipal or domestic sewage and nitrogenous waste are the sources of nitrate contamination in the study area. Groundwater sources have been affected by seepage along the Yamuna river and the apparent surface water–groundwater interactions. Excessive NO_3_^−^ in drinking water can cause some disorders including methemoglobinemia in infants, gastric cancer, goiter and hypertension in adults.^44^ Therefore, several researchers used various methods for its removal from groundwater.^45–47^ The fluoride content was higher than the guideline value designated by WHO^37^ and BIS^34^ in 64.28% of the samples. The highest concentration of 4.12 mg L^−1^ was reported at Shahganj, which has potential to cause fluorosis with long-term damage to the brain, liver, thyroid and kidneys.^48,49^ The spatial distribution of fluoride in the groundwater of the study area is shown in Fig. 5. The source of fluoride is mostly natural, from the disintegration of rocks and soils or the weathering of fluoride-bearing minerals such as florahalite ore and fluorite. However, there are also other sources of fluoride in groundwater such as industrial waste, municipal solid waste dumping and the seepage of untreated sewage water into the Yamuna river.

### 3.3. Concentration of trace metals in groundwater

Table 1 shows the mean concentration of different trace metals in groundwater samples along with other relevant statistical distribution parameters. The investigated trace metals in order of decreasing mean concentration were Fe > Zn > Mn > Cu. Iron concentrations spanned a wide range of 0.005–1.05 mg L^−1^, with an average value of 0.32 mg L^−1^. Iron primarily occurs naturally in soils, rocks and minerals, but some anthropogenic sources such as industrial effluents, sewage landfill leachate and the dissolution of iron from ferrous boreholes and hand pumps may also contribute to elevating the iron level in groundwater. The iron concentration exceeded the recommended BIS level in 39.28% of the samples. The highest concentration of 1.05 mg L^−1^ was observed at Sultanpura. The concentration of iron available in water does not threaten human health, but adverse health effects may occur due to chronic ingestion of high concentrations of iron.^50^ The concentration of Zn varied from 0.016–0.88 mg L^−1^ with an average value of 0.17 mg L^−1^. Zinc poisoning, which causes nausea, abdominal cramping, vomiting, tenesmus and diarrhea with or without bleeding, is associated with high levels of zinc concentration in drinking water.^51^ However, Zn concentrations were under the recommended limit designated by the BIS and WHO in all of the samples. The manganese concentration in the groundwater samples varied from BDL–0.51 mg L^−1^ (avg. 0.08 mg L^−1^). About 17.85% of the samples exceeded the acceptable limit (0.1 mg L^−1^) designated by BIS and WHO. The most common source of manganese in groundwater is the natural weathering of manganese-bearing minerals. Industrial effluents, sewage and landfill leachate are some anthropogenic sources which may raise manganese concentration in groundwater. Manganese does not threaten human health at a normal concentration in drinking water. However, a higher concentration of manganese may affect learning ability and intelligence quotient in children, while neurological damage, resulting in Parkinson’s-like symptoms, emotional liability and hallucinations are symptoms of manganese over-exposure in adults.^52^ Copper is an essential element for living organisms including humans, and it is necessary in small amounts in our diet to ensure good health. However, the excessive ingestion of Cu can cause serious toxicological concerns, such as vomiting, diarrhea, stomach cramps and nausea, or even death.^53^ The concentration of copper in the investigated samples varied from BDL–0.26 mg L^−1^ with an average of 0.018 mg L^−1^. The major sources of copper in groundwater are the corrosion of household plumbing systems and the erosion of natural deposits.^42^ The concentrations of copper were well within the permissible limits designated by the BIS and WHO standards. Thus, the groundwater in the studied area can be considered safe in terms of zinc and copper content.

### 3.4. Hydrochemical facies

**Fig. 6:**
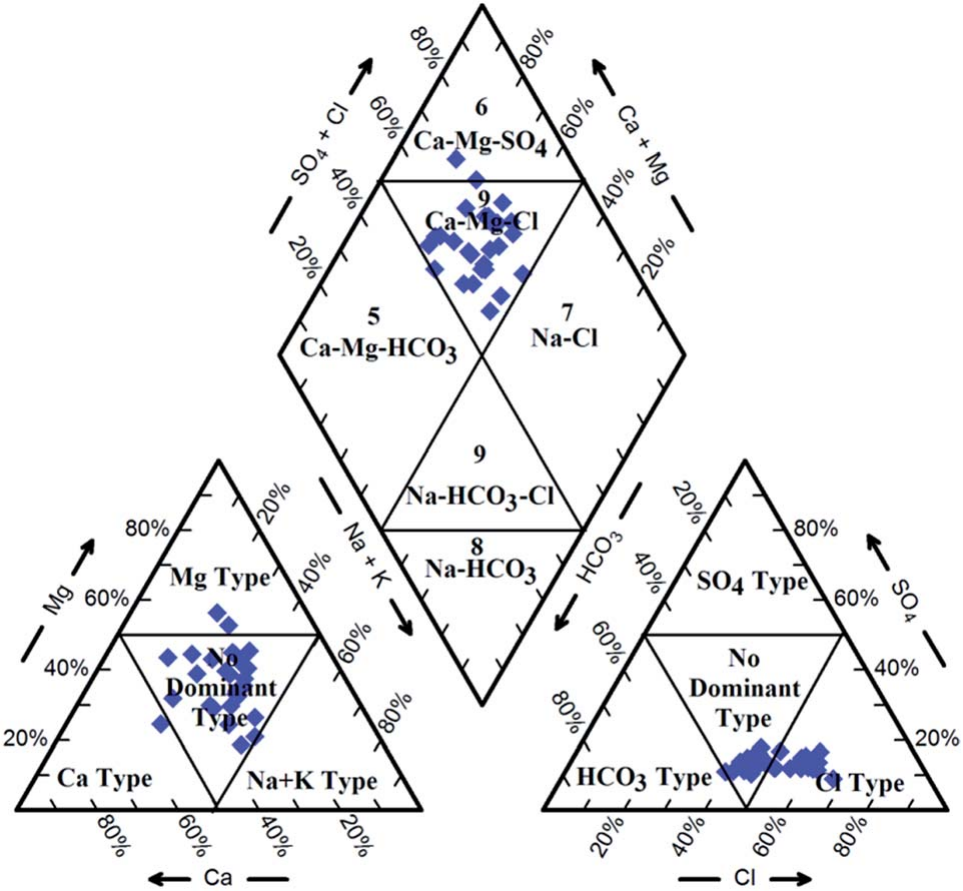
Piper trilinear diagram showing hydrogeochemical character and hydrochemical facies in the groundwater of Agra city.

**Fig. 7:**
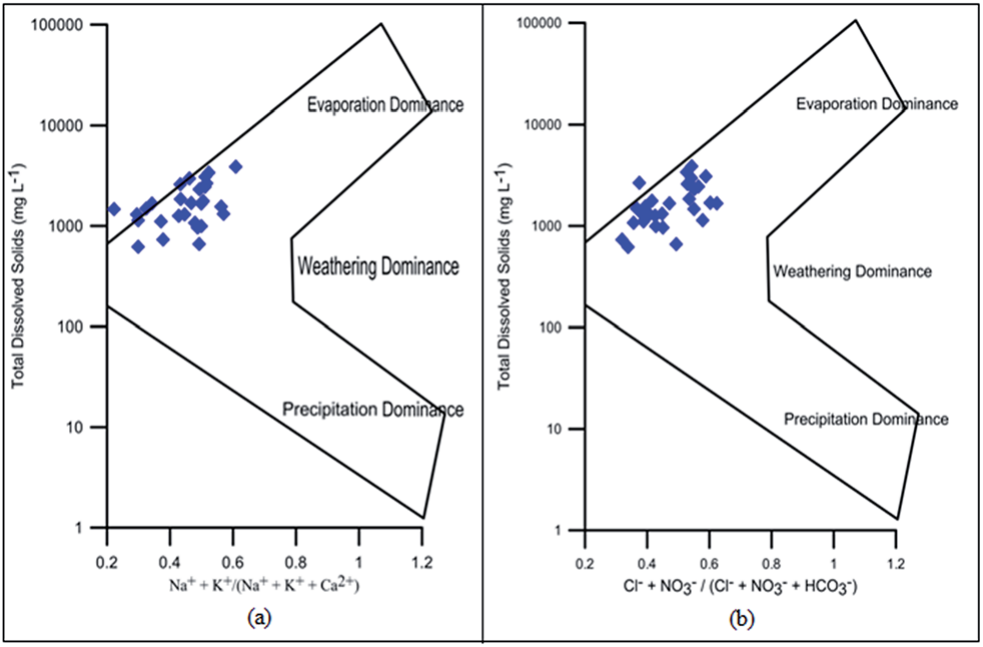
Gibbs diagram representing the ratio of (a) Na^+^ + K^+^/(Na^+^ + K^+^ + Ca^+^) and (b) Cl^−^ + NO_3_^−^/(Cl^−^ + NO_3_^−^ + HCO_3_^−^) as a function of TDS.

Hydrochemical facies can be defined as zones within a groundwater system with unique combinations of cation and anion concentrations.^54^ This concept is useful for developing a model to explain the genesis and distribution of principal groundwater types.^55^ The geochemical evolution of the groundwater and its relationship with different dissolved ions can be understood by plotting the geochemical data on a Piper^56^ trilinear diagram. The triangular cationic zone of the Piper diagram revealed that most of the groundwater samples (89%) fall into no dominant class. One of the samples was classified as a Ca^2+^ zone and two were classified as Mg^2+^ zones in the cationic triangle, whereas in the anionic triangle, about 50% of the samples fell into no dominant zone. The rest of the samples fell into the Cl^−^ zone in the anion triangle (Fig. 6). Moreover, the plotted points of 93% of the groundwater samples fell in zone 9, indicating an intermediate (mixed) chemical character of the groundwater, with none of the cation–anion pairs being dominant in the chemical composition. About 7% of the samples fell into zone 6, suggesting non-carbonate hardness. The characteristics of water in each zone of the Piper trilinear diagram are shown in Table 3. Based on the dominance of different cations and anions in the groundwater, a major hydrogeochemical water type in the study area can be defined as Ca^2+^–Mg^2+^–Cl^−^–SO_4_^2−^. A Gibbs diagram representing the ratio of Na^+^ + K^+^/(Na^+^ + K^+^ + Ca^2+^) and Cl^−^ + NO_3_^−^/(Cl^−^ + NO_3_^−^ + HCO_3_^−^) as a function of TDS can be used to understand the functional sources of dissolved chemical constituents, such as precipitation/rock/evaporation dominance.^57^ The plot of the geochemical data on Gibbs diagrams suggested rock weathering as a major driving force, with evaporation being a minor influence, thus controlling the groundwater chemistry of the study area (Fig. 7).

**Table 3:** Characteristics of water in each zone of the Piper trilinear diagram

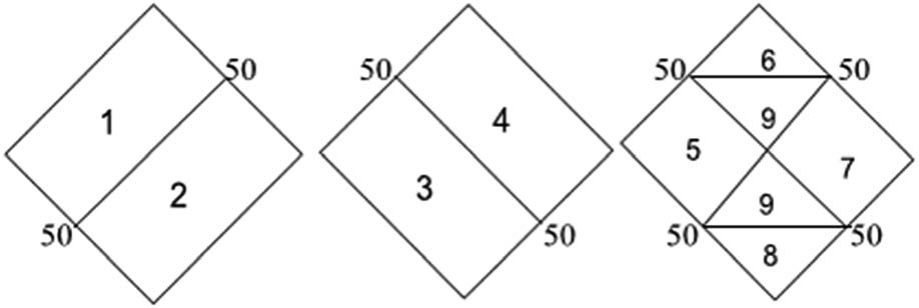	
Zone	Characteristics of water
1	Alkaline earth (Ca + Mg) exceeds alkali (Na + K)
2	Alkali exceeds alkaline earth
3	Weak acid (CO_3_ + HCO_3_) exceeds strong acid (SO_4_ + Cl)
4	Strong acid exceeds weak acid
5	Carbonate hardness (secondary alkalinity) exceeds 50%
6	Non-carbonate hardness (secondary salinity) exceeds 50%
7	Non-carbonate alkali (primary salinity) exceeds 50%
8	Carbonate alkali (primary alkalinity) exceeds 50%
9	No one cation–anion pair exceeds 50%

### 3.5. Correlation analysis of groundwater samples

Table 4 shows the statistical correlation matrix of various elements. Pearson correlation is a common statistical test used for determining the extent of association or correlation between two variables. In this study, there is a high correlation between various anions and cations due to anthropogenic activity in the surrounding area of the sampling site.

**Table 4:** Inter-elemental correlation matrix of dissolved ions (*n* = 28)

Parameter	pH	EC	DO	Turbidity	TDS	F^−^	Cl^−^	HCO_3_^−^	SO_4_^2−^	NO_3_^−^	Ca^2+^	Mg^2+^	Na^+^	K^+^	TH	Zn	Cu	Fe	Mn
pH	1.00																		
EC	0.34	1.00																	
DO	0.20	−0.07	1.00																
Turb	0.18	0.22	−0.02	1.00															
TDS	0.31	0.99	−0.03	0.23	1.00														
F^−^	0.37	0.32	−0.26	0.29	0.28	1.00													
Cl^−^	0.27	0.97	−0.06	0.13	0.96	0.21	1.00												
HCO_3_^−^	0.38	0.90	0.00	0.42	0.93	0.36	0.81	1.00											
SO_4_^2−^	0.30	0.94	0.04	0.08	0.97	0.23	0.92	0.87	1.00										
NO_3_^−^	0.37	0.94	−0.03	0.14	0.96	0.35	0.89	0.89	0.95	1.00									
Ca^2+^	0.25	0.88	−0.05	0.20	0.90	0.14	0.89	0.81	0.87	0.81	1.00								
Mg^2+^	0.28	0.80	0.09	0.27	0.78	0.39	0.74	0.72	0.75	0.84	0.57	1.00							
Na^+^	0.24	0.93	−0.10	0.20	0.96	0.24	0.92	0.89	0.92	0.89	0.84	0.62	1.00						
K^+^	0.24	0.93	0.02	0.29	0.93	0.18	0.89	0.87	0.87	0.88	0.76	0.76	0.91	1.00					
TH	0.30	0.95	0.03	0.27	0.94	0.31	0.91	0.86	0.91	0.93	0.86	0.91	0.81	0.86	1.00				
Zn	0.01	0.31	−0.16	0.09	0.33	0.04	0.33	0.28	0.29	0.38	0.16	0.40	0.30	0.34	0.33	1.00			
Cu	0.13	0.29	−0.26	0.19	0.30	0.23	0.12	0.45	0.31	0.42	0.22	0.30	0.27	0.35	0.30	0.20	1.00		
Fe	0.08	−0.15	−0.26	0.29	−0.17	0.30	−0.25	−0.03	−0.16	−0.12	−0.14	−0.21	−0.13	−0.16	−0.20	0.03	0.48	1.00	
Mn	0.20	0.16	−0.09	0.23	0.13	−0.10	0.08	0.21	0.10	0.16	0.08	0.19	0.12	0.25	0.16	0.28	0.52	0.35	1.00

**Fig. 8:**
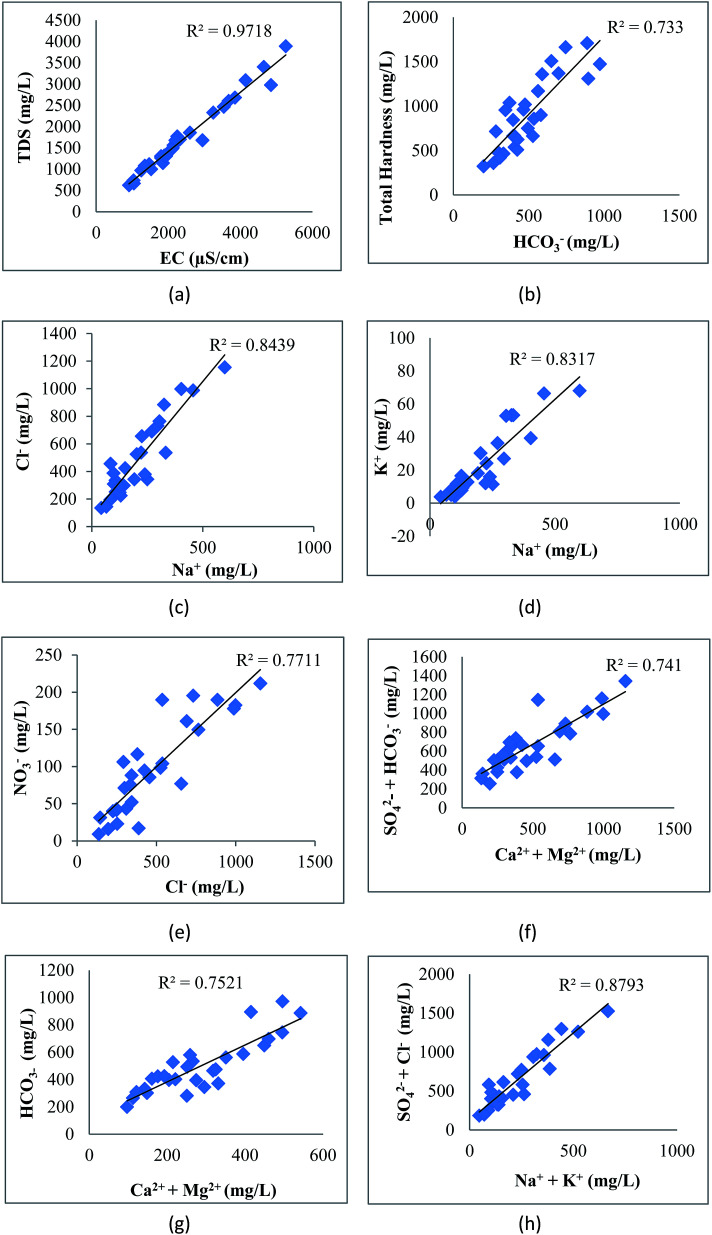
Correlation of (a) EC with TDS, (b) HCO_3_^−^ with TH, (c) Na^+^ with Cl^−^, (d) Na^+^ with K^+^, (e) Cl^−^ with NO_3_^−^, (f) Ca^2+^ + Mg^2+^ with HCO_3_^−^, (g) Ca^2+^ + Mg^2+^ with SO_4_^2−^ + HCO_3_^−^ and (h) Na^+^ + K^+^ with SO_4_^2−^ + Cl^−^.

The correlation of various elements is shown in Fig. 8. EC shows strong correlation with TDS (0.99), Cl^−^ (0.97), HCO_3_^−^ (0.90), SO_4_^2−^ (0.94), NO_3_^−^ (0.94), Ca^2+^ (0.88), Mg^2+^ (0.80), Na^+^ (0.93), K^+^ (0.93) and TH (0.95). The perfect correlation between EC and TDS indicated the high content of dissolved ions in the water. The total dissolved solids include organic and inorganic salts, such as Ca^2+^, Mg^2+^, Na^+^, K^+^, Cl^−^, HCO_3_^−^ and SO_4_^2−^. The correlation coefficient of TDS with Ca^2+^, Mg^2+^, Na^+^, K^+^, Cl^−^, HCO_3_^−^, SO_4_^2−^, NO_3_^−^ and TH is very high, showing the dissolution of salts in groundwater from anthropogenic sources such as industrial effluent and domestic discharge. This correlation of TDS with Na–HCO_3_–Cl or Na–HCO_3_–Mg may be due to the high fluoride concentration as studied by Deng *et al.*^58^ The positive correlation of total hardness with bicarbonate, calcium, and magnesium content shows that hardness is due to the presence of bicarbonate salts of calcium and magnesium. There is also a strong correlation of Cl^−^ with HCO_3_^−^, SO_4_^2−^, NO_3_^−^, Ca^2+^, Mg^2+^, Na^+^, K^+^ and TH; HCO_3_^−^ with SO_4_^2−^, NO_3_^−^, Ca^2+^, Mg^2+^, Na^+^, K^+^ and TH; SO_4_^2−^ with NO_3_^−^, Ca^2+^, Mg^2+^, Na^+^, K^+^ and TH; NO_3_^−^ with Ca^2+^, Mg^2+^, Na^+^, K^+^ and TH; Ca^2+^ with Na^+^, K^+^ and TH; Mg^2+^ with K^+^ and TH; Na^+^ with K^+^ and TH; and K^+^ with TH. These various correlations indicated that the process of weathering, exchangeable ions and reduction/oxidation, in conjunction with anthropogenic activity, may have caused the dissolution of salts in groundwater.^59^ Samantara *et al.*^42^ also observed a similar correlation between sulphate and chloride which might be due to the similar biochemical pathways that they follow. There is also a significant correlation between Ca^2+^ and Mg^2+^ and between Mg^2+^ and Na^+^.

### 3.6. Principal component analysis

Principal component analysis (PCA) is a statistical analysis technique to identify patterns of data to make it easy to explore. It involves multivariate analysis which transforms a large set of correlated variables into a small set of uncorrelated variables. The tool is based on covariance which represents the inter-relationships of the variable.^60^ It is also known as a dimensionless reduction tool because it constructs a new set of variables by reducing a large dataset. PCA can be used for the association of chemical compositions defined by one or more variable loadings on the factor that influences groundwater quality. A factor loading value close to ±1 indicates a strong correlation between the variables and the factor, while values >± 0.5 are considered significant.

**Fig. 9:**
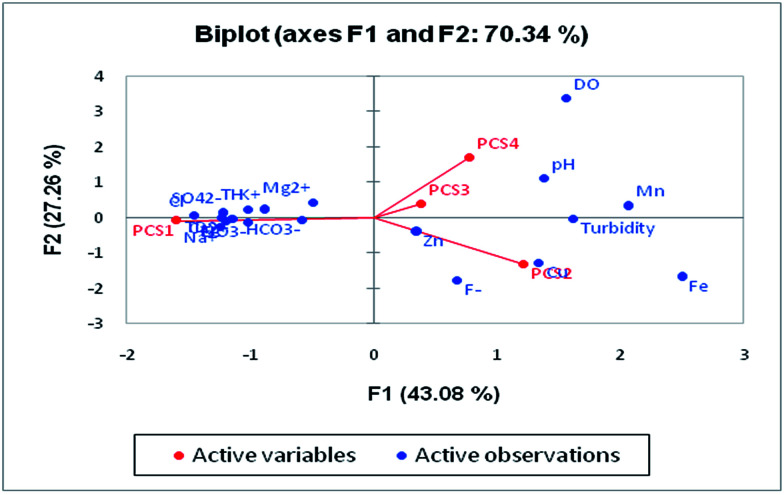
Loadings of the first four factors in the biplot showing the PCA of the water quality variables.

Four major eigenvalues (PC1, PC2, PC3 and PC4) were found in 28 groundwater samples for 19 parameters which could explain 79.95% of the variability. PC1 has the maximum variance in the data, followed by PC2, PC3 and PC4, respectively (Table 5). There is 54.25% of the variation in PC1 which exhibits significant loadings of EC, TDS, Cl^−^, HCO_3_^−^, SO_4_^2−^, NO_3_^−^, Ca^2+^, Mg^2+^, Na^+^, K^+^ and TH. PC1 mainly represented the major anions and cations resulting from natural and anthropogenic sources. The natural processes include water–rock interaction and the weathering of minerals in the aquifer,^61^ while the anthropogenic sources are attributed to industrial effluents, municipal solid waste and untreated sewage discharge. NO_3_^−^ loading is explained by onsite sanitation and nutrient contamination from an unsewered urban environment. PC2 was influenced by Cu and Fe and accounted for 12.03% of the total variance. The sources of these ions are anthropogenic activity in the study area. The high loading of Fe is due to the leaching of Fe-rich sediments such as laterites and lateritic soils into the groundwater. PC3 contributes 7.23% of the total variance with significant loadings of fluoride and pH which suggested that fluoride is influenced by pH. The leaching of fluoride from florahalite ore and the continuous dumping of untreated sewage into the Yamuna river is responsible for the significant loadings of fluoride. PC4 shows moderate loadings of DO, trace metals and Mn with a total variance of 6.43%. The presence of Mn in groundwater can be associated with untreated sewage and landfill leachate. Biplots of the first four components are shown in Fig. 9.

**Table 5:** Principal component analysis of groundwater samples in Agra city^a^

Variables	Component			
	PC1	PC2	PC3	PC4
pH	0.36	0.18	**0.59**	0.32
EC	**0.99**	−0.06	0.00	−0.06
DO	−0.03	−0.41	0.33	**0.73**
Turbidity	0.28	**0.45**	0.34	0.17
TDS	**0.99**	−0.09	−0.02	−0.04
F^−^	0.33	0.40	**0.58**	−0.46
Cl^−^	**0.94**	−0.22	−0.06	−0.09
HCO_3_^−^	**0.94**	0.12	0.10	0.04
SO_4_^2−^	**0.95**	−0.14	−0.04	−0.02
NO_3_^−^	**0.97**	0.00	−0.01	−0.03
Ca^2+^	**0.87**	−0.14	−0.03	−0.08
Mg^2+^	**0.83**	−0.01	0.07	0.12
Na^+^	**0.93**	−0.08	−0.08	−0.11
K^+^	**0.93**	−0.03	−0.11	0.09
TH	**0.96**	−0.08	0.03	0.03
Zn	0.36	0.19	−0.48	0.08
Cu	0.37	**0.71**	−0.22	0.05
Fe	−0.12	**0.83**	0.07	−0.08
Mn	0.20	0.60	−0.36	**0.52**
Eigenvalue (%)	10.30	2.28	1.37	1.22
% of variance	54.25	12.03	7.23	6.43
Cumulative %	54.25	66.28	73.51	79.95

a Highlighted values are considered as significant.

### 3.7. Evaluation and assessment of health risk due to trace metals

The dietary health risk was estimated for all of the investigated metals. The non-carcinogenic health risk in adults due to exposure to trace metals through ingestion is shown in Table 6. The ADD was calculated for minimum, maximum and mean concentrations of Zn, Cu, Fe and Mn. The average daily dose depends on the water consumption, weight and age of an individual. The HQ values for all trace metals were less than unity which indicated that these metals do not pose any adverse health effect to humans when groundwater in the studied areas is consumed by adults. The metals in order of decreasing HQ were Mn > Zn > Fe > Cu.

**Table 6:** Average daily dose and hazard quotient indices with reference dose for studied trace metals

Metal	Concentration (mg L^−1^)	ADD	RfD	HQ	HI
		(mg per kg per day)	(mg per kg per day)		
Zn	Min = 0.016	4.6 × 10^−4^	0.30	1.5 × 10^−3^	5.7 × 10^−2^
	Max = 0.880	2.5 × 10^−2^		8.3 × 10^−2^	
	Mean = 0.170	4.8 × 10^−3^		1.6 × 10^−2^	
Cu	Min = 0.000	0	0.04	0	
	Max = 0.260	7.4 × 10^−3^		0.18	
	Mean = 0.018	5.2 × 10^−4^		1.2 × 10^−2^	
Fe	Min = 0.005	1.4 × 10^−4^	0.70	2.0 × 10^−4^	
	Max = 1.050	3.0 × 10^−2^		4.3 × 10^−2^	
	Mean = 0.320	9.1 × 10^−3^		1.3 × 10^−2^	
Mn	Min = 0.004	1.2 × 10^−4^	0.14	8.2 × 10^−4^	
	Max = 0.510	1.4 × 10^−2^		0.10	
	Mean = 0.080	2.3 × 10^−3^		1.6 × 10^−2^	

The calculated hazard index across all metals served as a conservative assessment tool to estimate high-end risk rather than low-end risk in order to protect the public. This served as a screening value to determine whether the exposure to heavy metals in the groundwater may pose a significant health risk to the inhabitants. The estimated HI value was less than one, *i.e.* 5.7 × 10^−2^ (Table 6), therefore exposure to these elements through groundwater is not likely to exert a negative or cumulative adverse risk on the inhabitants in the study area.

### 3.8. Evaluation of groundwater quality using the water quality index (WQI)

**Fig. 10:**
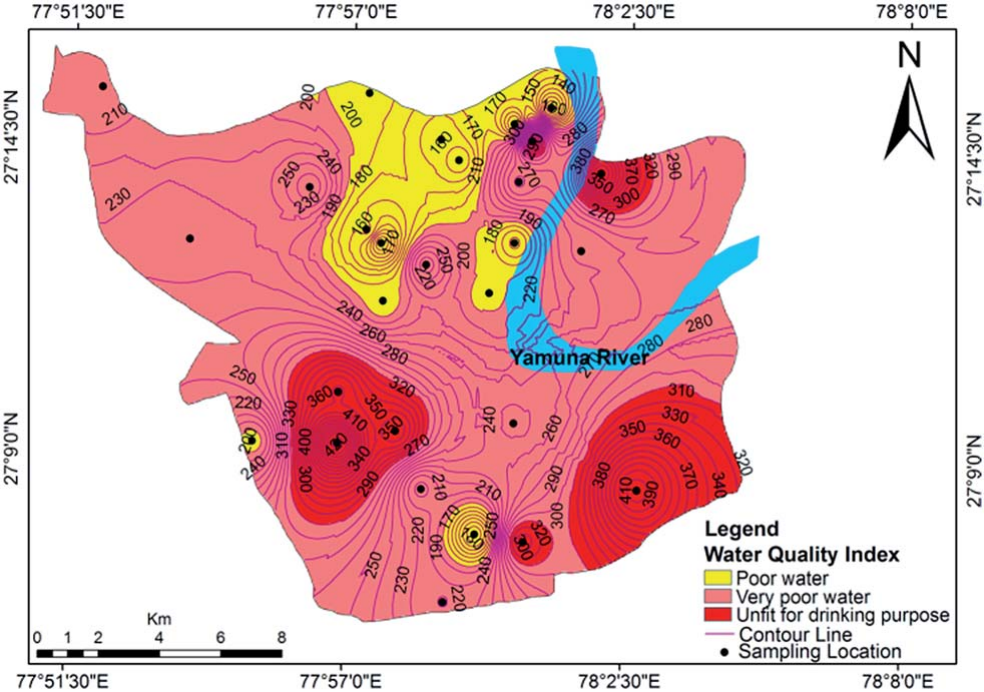
Water quality index map for groundwater in Agra.

The relative weights of the major components are computed and shown in Table 7. The computed WQI values were classified into different categories, as shown in Table 8. The WQI values at different locations are given in Table 9 and the spatial variation of the WQI is mapped in Fig. 10. The WQI values for groundwater in Agra city ranged from 109 to 455 with an average value of 240. The high values of WQI were mainly due to high TDS, F^−^, Cl^−^, NO_3_^−^, Mg^2+^, Na^+^ and TH. As per WQI categorization, the studied water samples fall under ‘poor’, ‘very poor’ and ‘unsuitable’ categories, with values of 46.42%, 28.57% and 25%, respectively. The groundwater at Langre ki Chowki, Agra Cantt, Namner, Shahganj, Balkeshwar, Rambagh, Tajganj and Sultanpura was unfit for drinking purposes. No sample was observed in ‘excellent’ or ‘good’ categories of groundwater quality. This indicated that the groundwater in the study area is unsafe for drinking purposes, and hence its remediation and treatment is necessary prior to human consumption.

**Table 7:** Relative weights of the major components^a^

Chemical parameters	Standards (BIS 2012)	Weight (*w*_*i*_)	Relative weight (*W*_*i*_)
pH	8.5	4	0.07
Turbidity	5	4	0.07
Total dissolved solids (TDS)	500	5	0.08
Fluoride (F^−^)	1.5	5	0.08
Chloride (Cl^−^)	250	5	0.08
Nitrate (NO_3_^−^)	50	5	0.08
Sulphate (SO_4_^2−^)	200	5	0.08
Bicarbonate (HCO_3_^−^)	200	1	0.02
Calcium (Ca^2+^)	75	3	0.05
Magnesium (Mg^2+^)	30	3	0.05
Total hardness (TH)	100	2	0.03
Zinc (Zn)	5	4	0.07
Copper (Cu)	0.05	5	0.08
Iron (Fe)	0.3	4	0.07
Manganese (Mn)	0.1	5	0.083
		Σ*w*_*i*_ = 60	Σ*W*_*i*_ = 1.00

a All parameters are in mg L^−1^ except for pH and turbidity.

**Table 8:** Classification of water quality based on the WQI range and the % of samples in each respective category^29,33,62^

WQI range	Type of water	% of samples
<50	Excellent water	Nil
50–100	Good water	Nil
100–200	Poor water	46.42
200–300	Very poor water	28.57
>300	Unfit for drinking purposes	25

**Table 9:** Water quality index (WQI) values of groundwater in Agra

S. no.	Place name	Source of water	Latitude	Longitude	WQI	Description
1	Sikandra	Hand pump	27.25°	77.86°	207	Very poor
2	Khandari	Hand pump	27.22°	77.93°	256	Very poor
3	Dayal Bagh	Hand pump	27.25°	77.95°	194	Poor water
4	Langre ki Chowki	Tube well	27.23°	78.00°	272	Very poor
5	Balkeshwar	Hand pump	27.24°	78.00°	365	Unsuitable
6	Rambagh	Hand pump	27.23°	78.03°	410	Unsuitable
7	Belanganj	Hand pump	27.21°	78.00°	167	Very poor
8	Daresi	Hand pump	27.19°	77.99°	194	Poor water
9	Shahganj	Hand pump	27.16°	77.94°	361	Unsuitable
10	Agra Cantt	Tube well	27.12°	78.00°	325	Unsuitable
11	Baluganj	Hand pump	27.16°	78.00°	237	Poor water
12	Tajganj	Hand pump	27.14°	78.04°	414	Unsuitable
13	Bundu Katra	Hand pump	27.10°	77.98°	219	Poor water
14	Loha Mandi	Hand pump	27.21°	77.89°	237	Poor water
15	Kamla Nagar	Hand pump	27.24°	78.00°	135	Poor water
16	Hariparwat	Hand pump	27.21°	77.95°	172	Poor water
17	St. John’s College	Hand pump	27.21°	77.96°	109	Poor water
18	Suresh Nagar	Tube well	27.24°	77.97°	160	Very poor
19	Itmad-ud-Daula	Tube well	27.21°	78.02°	251	Very poor
20	Sadar Bazar	Hand pump	27.14°	77.97°	206	Very poor
21	Sultanpura	Hand pump	27.15°	77.94°	455	Unsuitable
22	Gandhi Nagar	Hand pump	27.23°	77.98°	151	Poor water
23	Nai Ki Mandi	Hand pump	27.19°	77.96°	190	Poor water
24	Mantola	Tube well	27.20°	77.97°	256	Very poor
25	Rajwara	Tube well	27.25°	78.01°	110	Poor water
26	Namner	Hand pump	27.15°	77.96°	357	Unsuitable
27	Naulakha	Hand pump	27.12°	77.99°	131	Poor water
28	Idgah Colony	Hand pump	27.15°	77.91°	194	Poor water

## 4. Conclusions

Groundwater quality was determined in the present study at different locations in Agra city for drinking purposes. The findings of this study concluded that the groundwater in the studied area is unsuitable for drinking purposes. The various physicochemical parameters of most of the groundwater samples exceeded the BIS and WHO permissible limits for drinking water, which may substantially harm the health of the residents in the area. Anthropogenic sources such as industrial waste, untreated sewage water, municipal solid waste dumping and automobile emissions might be the factors causing the excessive concentration of various parameters. The cationic concentrations of Mg^2+^ and Na^+^ as well as the anionic concentrations of HCO_3_^−^ and Cl^−^ are dominant in the groundwater. The groundwater is laden with an objectionable concentration of cations and anions which may have been derived from a number of different sources, *i.e.*mineralization, the chemical weathering of rock, mine tailings and sewage contamination. Gibbs diagrams suggest rock weathering as a major driving force along with evaporation as a minor influence, thus controlling the groundwater chemistry. The concentrations of the studied trace metals (Zn, Fe, Cu and Mn) in the groundwater samples complied with the WHO and BIS standards for drinking water. The value of the hazard index was 5.7 × 10^−2^ for trace metals, which is much less than 1, indicating that there will be no potential health effects from trace metals. On the basis of the water quality index, almost half of the samples belong to the ‘poor’ category and the other half of the samples fall in the ‘very poor’ and ‘unfit for drinking purposes’ categories. Therefore, appropriate treatment and remediation techniques are required prior to human consumption. Spatial distribution maps communicated possible information regarding the overall water quality distribution in the study area, and they are a useful technique for monitoring, management and future modeling with the aid of a GIS tool. This study strongly recommends continuous groundwater monitoring in and around the study area for planning and implementation in order to meet water supply demand without compromising the ability of future generations to meet water quality requirements.

## Conflicts of interest

There are no conflicts of interest to declare.

## Supplementary Material
